# The Influence of Sperm Morphology, Total Motile Sperm Count of Semen and the Number of Motile Sperm Inseminated in Sperm Samples on the Success of Intrauterine Insemination

**Published:** 2011-12-22

**Authors:** Roshan Nikbakht, Nasrin Saharkhiz

**Affiliations:** Fertility and Infertility Research Center, Ahvaz Jundishapur University of Medical Science, Ahvaz, Iran

**Keywords:** Intrauterine, Insemination, Motile Sperm, Morphology

## Abstract

**Background:**

The present study aimed to analyze the prognostic value of sperm morphology , total motile sperm count (TMSC) and the number of motile sperm inseminated (NMSI) on the outcome of intrauterine insemination (IUI).

**Materials and Methods:**

This cross sectional study was carried out 445 women undergoing 820 IUI cycles. All of the patients underwent controlled ovarian hyper stimulation with clomiphen citrate and human menopausal gonadotropin (HMG) followed by intrauterine insemination with the husband’s sperm. Pregnancy rate (PR) per cycle in correlation to sperm morphology, TMSC and NMSI was obtained. Statistical analysis of the data was done by the SPSS version 13 (Chicago,USA).

**Results:**

A total of 81 clinical pregnancies were obtained for a pregnancy rate per cycle of 9.9%. When the TMSC was 5×10^6^to <10×10^6^, the PR per cycle was significantly higher than the subgroups <1×10^6^, 1×10^6^to <5×10^6^and ≥10×10^6^(15%, 5.6%, 5.1%, 10.8%, respectively). Sperm morphology was in itself a significant factor that affected the likelihood of IUI success. Nonetheless, the most significant difference of the PR per cycle with sperm morphology was in the subgroup <5 % (2.1% vs. 97.9%).When the NMSI was ≥10×10^6^, the PR per cycle was significantly higher than the subgroups<5×10^6^and 5×10^6^to< 10× 10^6^(11.2%, 4.1%, 5.2%, respectively).

**Conclusion:**

The study showed that TMSC 5×10^6^to < 10×10^6^and normal sperm morphology ≥ 5% and NMSI ≥ 10×10^6^are useful prognostic factors of IUI cycles.

## Introduction

Artificial insemination has been used to treat infertile couples for almost 200 years. Intrauterine insemination (IUI) is now performed for several reasons. The cut-off level of semen parameters in predicting the likelihood of successful IUI is still unequivocal (1-5). It is not determined which parameter of semen is essential for diagnosis in couples who will benefit from IUI (6).

Some pregnancy will occur after IUI even with sever male factor. Clinicians need tests that identify which sub-fertile couples are likely to benefit from IUI (7).

The effectiveness of IUI depends mainly on semen quality, which is assessed by the total motile sperm count (TMSC) and sperm morphology.

TMSC in the ejaculate is the product of multiplying the semen volume by the sperm concentration by the percentage of progressively motile sperms. The best results are achieved when the number of TMSC exceeds a threshold of approximately 10 million (1, 3-5).

Sperm morphology is another factor that may influence the IUI result. Most studies have found a strong correlation between sperm morphology and the IUI result. In assessing sperms morphology by strict criteria, success rates with IUI are highest when 14% or more of the sperm have normal morphology, like the results observed in *in vitro* fertilization (IVF) cycles (1, 8-11).

The post-wash total motile count (TMC) has been proposed as a test to help distinguish the couples who would benefit from IUI, but it could not distinguish between the couples who are likely to benefit from IUI and those more likely to benefit from IVF or intracytoplasmic sperm injection (ICSI) (7). Several studies have shown the increase of pregnancy rate after IUI when the number of motile sperm inseminated (NMSI) was between 0.8×10^6^ to 20×10^6^ (12-14) .

The aim of our study was to assess the threshold of TMSC, sperm morphology and NMSI on the IUI outcome.

## Materials and Methods

This study was a cross sectional. Four hundred forty five couples completed 820 IUI cycles in the infertility department of Imam khomieni Hospital in Ahvaz, Iran from May 2004 to May 2006. They were candidates for IUI because of male factor infertility or unexplained infertility. Informed consent forms were signed by all patients. This study was approved by the Ethics Committee of Ahvaz Jundishapour University Medical Sciences.

Inclusion criteria were normal thyroid stimulating hormone (TSH), prolactin levels and hysterosalpingography. Laparoscopy was performed for suspicious tubal and peritoneal factors before any treatment.

Serological tests human immunodeficiency virus (HIV) antibody, hepatitis B surface antigen (HbsAg) and hepatitis C virus (HCV) antibody were conducted for all the couples.

At first, all women were examined by the vaginal ultrasound (Honda 2000, 7.5 MHZ Transducer, Japan) on 1-5th days of their menstrual period to ensure that ovarian follicles were smaller than 15 mm.Then they underwent controlled ovarian hyperstimulation and received clomiphen citrate100 to 150 mg on the 3-5th day of cycle for 5days and at least 75IU HMG after the last dose of clomiphen citrate irrespective of whether they were ovulatory or anovulatory. Ovarian response was monitored by the vaginal ultrasound;when the follicular size of the leading follicle was 18- 22 mm, human chorionic gonadotropin (HCG) (5000 IU) was administered. All semen samples were collected in the laboratory after 2-3 days of sexual abstinence.

After liquefaction, the sperm volume, pH, count, motility and morphology were evaluated according to the WHO guidelines 1999 (15).

Raw semen was processed for IUI using swimup technique. The sampls were liquidated at 37°C and centrifuged at 300-500 g for 5-10 minutes. Then the supernants were discarded, the pellets were resuspended in 2 ml of medium (Ham’s F10 media, Steinheim, Germany) and centrifuged two times. In each time, the supernants were discarded. Finally, the pellets were resuspended in 0.5-1 ml of medium and the tubes were left at 37°C for 30-60 minutes in a humidified incubator to allow sperm to swimp up. Then the washed sperms were inseminated with an IUI catheter. No drug was used for luteal phase support.

Serum HCG levels were determined two weeks after the HCG injection in the absence of menstruation for diagnosis of pregnancy. A clinical pregnancy was defined as serum positive β-HCG.

The principal assessment criterion consists of the pregnancy rate per cycle according to TMSC, sperm morphology and NMSI.

### Statistical analysis

Statistical analysis of the data was done by the SPSS software (version 13, SPSS, Chicago,USA). The data were expressed as the mean standard deviation, independent t test and χ2 test. Odd ratios were calculated using the Logistic regression model for comparison of categorical variables. Significance was set at p<0.05.

## Results

In this study, 445 couples, who underwent 820 IUI treatment procedures ,were recruited. Demographic characteristics of the couples are listed in table 1.

**Table 1:** Profile of patients (distribution of variables)


	IUI outcome		
Variables	Positive	Negative	P

**Female age (years)**	27.58 ± 5.1	28.58 ± 5.2	0.105
**Male age (years)**	33.96 ± 7.8	33.65 ± 6.2	0.675
**Duration of infertility (months)**	62.83 ± 39	74.73 ± 50	0.042


Values are mean ± SD (95% confidence interval).

The range of female and male age were between "16 to 46" and "21 to 63" years and duration of infertility was 12-456 months.

**Table 2:** The results of IUI according to the age of women, kind of infertility and ovulation


	IUI outcome			
	Positive	Negative	P	X^2^

**Age of women (year)**			0.532	1.262
**Age ≤30**	54 (10.5)	458 (89.5)		
**30 ≤ Age <35**	18 (9.9)	163 (90.1)		
**Age ≥ 35**	9 (7.2)	116 (92.8)		
**Kind of infertility**			0.407	0.686
**Primary infertility, n (%)**	56 (6.89)	546 (67.24)		
**Secondary infertility, n (%)**	24 (2.95)	189 (23.27)		
**Ovulation**			0.001	10.744
**Positive**	43 (5.4)	523 (65.7)		
**Negative**	35 (4.39)	195 (24.49)		


Parentheses indicate the percentage

**Table 3:** The results of IUI according to TMSC , Normal sperm morphology and NMSI


	IUI outcome			
	Positive	Negative	P	X^2^

**TMSC (×10^6^)**			0.001	15.813
**n<1**	3 (5.6)	51 (94.4)		
**1 ≤ n<5**	14 (5.1)	258 (94.9)		
**5 ≤n<10**	39 (15)	221(85)		
**n≥10**	25 (10.8)	206 (89.2)		
**Normal sperm morphology (%)**	0.017	8.168		
**5<**			2 (2.1)	94 (97.9)
**5 ≤ n<10 **	54 (10.1)	482 (89.9)		
**≥10**	23 (12.6)	160 (87.4)		
**NMSI**	0.026	7.311		
**n<5×10^6^**			3(4.1)	70 (95.9)
**5x10^6^≤n<10×10^6^**	7(5.2)	128 (94.8)		
**n≥10×10^6^**	65 (11.2)	516 (88.8)		


Parentheses indicate the percentage

Seventy -three point eighty six percent and 26.13% of the couples had primary and secondary infertility, respectively.

Eighty one pregnancies followed 820 IUI cycles, and the total pregnancy rate per cycle was 9.9 %. There was a statistically significant difference between the clinical pregnancy rate and duration of infertility and ovulatory cycles (p=0.042, p=0.001, respectively) , but not with age of women and men and kind of infertility (Table 2).

Table 3 shows the results of IUI with TMSC. When the TMSC is 5×10^6^ to < 10×10^6^ , pregnancy rate is significantly higher than the subgroups with <1×10^6^, 1×10^6^ to <5×10^6^ and ≥10×10^6^ (15%, 5.6%, 5.1% and 10.8%, respectively) (p=0.001). By considering the clinical pregnancy rate according to normal sperm morphology, the most positive IUI cycles were observed in the subgroups with normal sperm morphology (5% or more) and the most difference of the results was in the subgroups with normal sperm morphology<%5 (2.1% vs. 97.9 %) (p=0.017), (Table 3).

Table 3 also indicates the results of IUI with the number of motile sperms inseminated. The PR per cycle was significantly higher when the number of motile sperms inseminated (NMSI) ≥10×10^6^ in comparable with the subgroups <5×10^6^ and 5×10^6^ to <10×10^6^ (11.2%, 4.1% and 5.2%, respectively). The difference is statistically significant (p =0.026).

On the other hand when the NMIS was divided into two groups of ≥10×10^6^ and <10×10^6^, according to Logistic regression model, the rate of pregnancy was higher in the first group (p=0.001, OR=2.86; CI, 1.57-5.21).

## Discussion

According to the findings of the present study, 81 clinical pregnancies were achieved after 820 IUI cycles for a total pregnancy rate per cycle of 9.9%. This rate is within the range of the previous studies (1, 3, 5, 7, 8, 16-18).

Overall, most of the previous studies have indicated that the female age, duration of infertility and ovulation are prognostic factors for IUI success (1). In this research, the duration of infertility was a prognostic factor but the female age was not. It may be due to the mean of female age in the two groups, which was approximately the same and lower than 35 years. Basirat et al. (18) reported that the female age and duration of infertility were correlated with the occurrence of pregnancy but the etiology of infertility, type of treatment regimen and the number of dominant follicles did not correlate with the pregnancy occurrence in an IUI cycle. Van Voorlis et al. (9) claimed that duration of infertility and infertility diagnosis in the women were not prognostic factors.

In the current study, we found that the most of IUI success when the range of total motile count was 5×10^6^ to <10×10^6^. Also, the findings of this study showed that TMC<1×10^6^ was not justified for IUI treatment.

In accordance with the present results, some previous studies have suggested that using the total number of motile spermatozoa of semen was a criteria for choosing between IUI and IVF and have recommended the threshold values of 5 to 10×10^6^ (1, 3-5, 8), but Akanji et al. (19) and Dorjpurev et al. (20) suggested IUI is possible in a condition that TMSC is greater than 10 million.

Sperm morphology is another factor that may influence the IUI results. It is worth mentioning that morphological assessment may vary substantially according to the condition of observation, and the kind of sperm morphology assessment, but like the results observed in IVF cycles, the probability of IUI success rises with the percentage of morphologically normal sperms. A number of prior studies have reported that IUI success rates are higher when 14% or more of the sperms have normal morphology and inseminated with the values between 4% and 14% and generally quite poor when fewer than 4% of sperms are normal (21-23).

In agreement with the above studies, the results of the present study also showed that when sperm morphology is more than 5%, the likelihood of IUI success is higher than when it is less than 5%.

Regarding the NMIS, as a factor that may influence on the IUI success, our finding showed that 11.2 % of the positive results were in the group that their NIMS was 10×10^6^ or more. On the other hand, rate of pregnancy after IUI was 2.86 times when NMIS ≥10×10^6^. This finding is in agreement with the study of Miller et al. (8).

Berg et al. also found a nonlinear increase in the PR per cycle with the increasing of NMIS in the uterine. They observed that insemination with <0.8×10^6^ motile sperms after swim-up resulted in a PR of <1% per treatment cycle. But when the motile sperm count was above this level, the PR per cycle reached a plateau of 6.9% to 10.2% (24).

Van weert et al. listed 16 studies reporting that at cut-off levels of 0.8 to 5 million motile spermatozoa, the post wash TMC provided a substantial discriminative performance. At these cut-off levels, the specificity of the post wash TMC was as high as 100% and the sensitivity of the test was limited (7).

Tay et al. (25) identified that PR was significantly lower in patient with NMSI ≤20 million /ml compared to those with TMC >20 million /ml.

Dadkhah et al. (26) also found that mean of total sperms after processing was significantly higher in IUI cycles with positive results.

However, Motazedian et al. (27) declared that there was no significant difference in the IUI outcome when normal sperm morphology is more than 20% or less than 20%. Dorjpurev et al. (20) and Burr et al. (28) indicated that number of motile sperm inseminated did not significantly affect the PR as well.

## Conclusion

The results of the present study identified a statistically significant difference in the TMSC, sperm morphology and the NMSI on the outcome of intra uterine insemination.
